# Achados Inesperados na Ressonância Magnética Cardíaca: Trombo Intracardíaco de Rápido Desenvolvimento com Fibrose Endomiocárdica

**DOI:** 10.36660/abc.20250030

**Published:** 2025-07-08

**Authors:** İrem Bilge Bulburu, Muhammet Gürdoğan, Fethi Emre Ustabaşıoğlu, Kenan Yalta

**Affiliations:** 1 Trakya University School of Medicine Department of Cardiology Edirne Turquia Department of Cardiology, School of Medicine, Trakya University, Edirne - Turquia; 2 Trakya University School of Medicine Department of Radiology Edirne Turquia Department of Radiology, School of Medicine, Trakya University, Edirne - Turquia

**Keywords:** Cardio-Oncologia, Ecocardiografia, Fibrose Endomiocárdica

## Introdução

A fibrose endomiocárdica (EMF) é uma forma rara de cardiomiopatia restritiva, particularmente prevalente em regiões tropicais. Essa condição é comumente associada à hipereosinofilia no sangue periférico, embora também possa ocorrer na ausência desse achado. Neste relato, descrevemos um caso incomum de EMF associado ao linfoma não Hodgkin sem hipereosinofilia, acompanhado por um trombo intracardíaco de rápido desenvolvimento.^1^

## Relato de Caso

Uma mulher de 25 anos com histórico de linfoma não Hodgkin apresentou-se ao departamento de emergência com dispneia de início recente. A paciente havia passado por quatro ciclos de quimioterapia contendo antraciclinas, sendo a sessão mais recente realizada dois meses antes da admissão.

**Figura 1:**
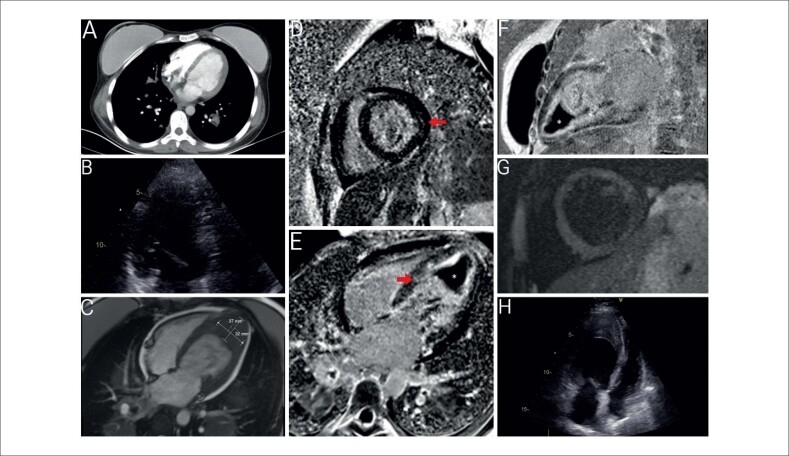
A) Imagem de Angiotomografia Computadorizada realizada no primeiro dia; não se observou trombo no ventrículo esquerdo (VE); B) Primeira imagem de Ecocardiografia Transtorácica (ETT) em quatro câmaras, sem trombo no VE; C) Imagem de Ressonância Magnética Cardíaca (RMC) em quatro câmaras mostrando trombo; D) Imagem de RMC em eixo curto mostrando Realce Tardio pelo Gadolínio (RTG) no miocárdio (seta vermelha); E) Imagem de RMC em quatro câmaras mostrando RTG subendocárdico e miocárdico (marcado com uma seta vermelha e * indicando o trombo); F) Imagem de RMC sagital mostrando RTG subendocárdico e miocárdico e * indicando o trombo; G) Imagem de RMC de recuperação de inversão de tau curto (STIR) em eixo curto mostrando aumento de sinal consistente com inflamação miocárdica; H) Imagem de ETT em quatro câmaras mostrando um defeito de preenchimento sugestivo de trombo detectado no ápice do VE.

Os exames de sangue revelaram um nível elevado de D-dímero (5,71 mg/L), troponina aumentada (231,8 ng/L), proteína C reativa elevada (11 mg/L) e uma contagem de eosinófilos marcadamente baixa (0,01×10^9^/L). A angiotomografia computadorizada realizada no departamento de emergência revelou um defeito de preenchimento no ramo segmentar medial da artéria pulmonar direita, sugestivo de embolia, enquanto nenhuma anormalidade ou trombo foi observado nas câmaras cardíacas ( Figura 1A ). A heparina de baixo peso molecular foi administrada duas vezes ao dia para embolia pulmonar.

A ecocardiografia transtorácica (ETT) de rotina revelou uma fração de ejeção (FE) de 55%, disfunção diastólica de grau 1 e nenhuma evidência de patologia ventricular ( Figura B ). A angiografia coronária foi realizada para excluir etiologia isquêmica, e a ressonância magnética cardíaca (RMC) foi planejada para descartar miocardite. As artérias coronárias pareceram normais.

Quatro dias após a avaliação ecocardiográfica e dez dias após o início da anticoagulação, a RMC revelou um trombo inesperado preenchendo o ventrículo esquerdo (VE), do ápice até a região média do ventrículo ( Figura 1C e Material Suplementar 1 ). Observou-se realce tardio do contraste consistente com EMF, sendo mais proeminente na região subendocárdica ( Figuras 1D, E e F ). Ainda, aumentos de sinal compatíveis com inflamação miocárdica e edema foram identificados nas imagens de recuperação de inversão de tau curto (STIR) ( Figura 1G ). Foi observado um derrame pericárdico de 17 mm. A ecocardiografia de acompanhamento confirmou a presença de trombo no ápice do VE ( Figura 1H , Material Suplementar 2 ).

O diagnóstico foi de FEM secundária à quimioterapia com antraciclinas, com formação de trombo intracardíaco como complicação da EMF. A síndrome de Loeffler foi descartada, pois os níveis de eosinófilos da paciente permaneceram no limite inferior da faixa normal. A paciente foi tratada com terapia antibiótica e anticoagulação, sem complicações embólicas observadas. Infelizmente, a paciente acabou sucumbindo a choque séptico e falência de múltiplos órgãos.

## Discussão

As taxas de sobrevivência aumentaram com os avanços nas terapias contra o câncer, mas isso também levou a uma maior incidência de efeitos colaterais cardíacos associados à quimioterapia.^2^ Consequentemente, a avaliação cardíaca de rotina e o monitoramento da FE são recomendados para pacientes com câncer, dependendo do regime específico de quimioterapia.^3^ Embora a ETT seja comumente usada para monitorar a função cardíaca, a RMC demonstrou ser mais sensível na detecção precoce de alterações na FE.^4^ Ela pode identificar reduções na FE antes que se tornem aparentes na ecocardiografia e detectar edema miocárdico e aumento do volume extracelular por meio das técnicas de mapeamento T1 e T2. Esses métodos permitem um diagnóstico mais precoce, muitas vezes antes do aparecimento da fibrose e do realce tardio pelo gadolínio, tornando a RMC uma ferramenta valiosa no manejo das complicações cardíacas relacionadas à quimioterapia, como o declínio da função ventricular esquerda associado à terapia com antraciclinas.^5^

Este caso destaca um trombo intracardíaco de desenvolvimento rápido em um paciente com câncer sob terapia anticoagulante. Embora o realce tardio do contraste na RMC seja tipicamente associado a danos miocárdicos irreversíveis, como os observados na doença arterial coronariana, o envolvimento de todo o subendocárdio, em vez de uma lesão localizada na região epicárdica da artéria coronária – junto com uma angiografia coronária normal – sugere que a FEM seja a doença primária.^6^ Estudos anteriores indicaram que as antraciclinas podem contribuir para o desenvolvimento de FEM cardíaca, dependendo do histórico de tratamento do paciente.^7^

O gatilho exato para a formação de trombos nesses pacientes ainda não está claro; no entanto, vários fatores, incluindo cardiotoxicidade induzida pela quimioterapia, um estado de hipercoagulabilidade e o desenvolvimento de FEM, provavelmente contribuem para a rápida formação de trombos intracavitários, o que pode levar a uma descompensação rápida e à morte.^3^ Portanto, os clínicos devem estar atentos ao reconhecimento da natureza multifatorial do estado de hipercoagulabilidade em pacientes com câncer, pois isso pode resultar no desenvolvimento acelerado de trombos intracavitários.^8^

A realização precoce de exames cardíacos de imagem deve ser considerada para pacientes com câncer que apresentem sintomas cardíacos ou pulmonares de início recente. A formação de trombos intracavitários deve ser monitorada de perto, particularmente em pacientes com múltiplos fatores de risco par hipercoagulação, que podem levar à mortalidade e à morbidade importantes.

Disponibilidade de Dados

Os conteúdos subjacentes ao texto da pesquisa estão contidos no manuscrito.

## *Material suplementar

Para assistir ao vídeo suplementar 1, por favor, clique aqui .

Video 1

Para assistir ao vídeo suplementar 2, por favor, clique aqui .

Video 2
