# Supplementary material for: Achados Inesperados na Ressonância Magnética Cardíaca: Trombo Intracardíaco de Rápido Desenvolvimento com Fibrose Endomiocárdica
Source: Arq Bras Cardiol. 2025 Jul 8;122(6):e20250030. [Article in Portuguese] doi: 10.36660/abc.20250030 (PMC12269913; doi:10.36660/abc.20250030)
Supplement: Translation

# Translation: Surprising Cardiac Magnetic Resonance Imaging Findings: Rapidly Developing Intracardiac Thrombus with Endomyocardial Fibrosis

**DOI:** 10.36660/abc.20250030i

**Authors:** İrem Bilge Bulburu, Muhammet Gürdoğan, Fethi Emre Ustabaşıoğlu, Kenan Yalta

**Affiliations:** 1 Edirne Türkiye Department of Cardiology, School of Medicine, Trakya University, Edirne – Türkiye; 2 Edirne Türkiye Department of Radiology, School of Medicine, Trakya University, Edirne – Türkiye

**Keywords:** Cardio-Oncology, Echocardiography, Endomyocardial Fibrosis

## Introduction

Endomyocardial fibrosis (EMF) is a rare form of restrictive cardiomyopathy, particularly prevalent in tropical regions.^1^This condition is commonly associated with peripheral blood hypereosinophilia, though it may also occur in the absence of such finding. In this report, we describe an unusual case of EMF linked to non-Hodgkin's lymphoma without hypereosinophilia, accompanied by a rapidly developing intracardiac thrombus.^1^

## Case Report

A 25-year-old woman with a history of non-Hodgkin's lymphoma presented to the emergency department with new-onset dyspnea. The patient had undergone four cycles of chemotherapy containing anthracyclines, with the most recent session occurring two months prior to admission.

Blood tests revealed an elevated D-dimer level (5.71 mg/L), increased troponin (231.8 ng/L), elevated C-reactive protein (11 mg/L), and a markedly low eosinophil count (0.01×10^9^/L). Computed tomography angiography (CTA) performed in the emergency department revealed a filling defect in the medial segmental branch of the right pulmonary artery, suggestive of an embolism, while no abnormalities or thrombus were observed in the heart chambers ( Figure 1A ). Low-molecular-weight heparin was administered twice daily for pulmonary embolism.

**Figure 1:** A) Computed tomography angiography image on the first day, no thrombus was observed in the left ventricle (LV); B) First transthoracic echocardiography (TTE) four-chamber view, no thrombus in the LV; C) Cardiac magnetic resonance (CMR) four-chamber view demonstrating thrombus; D) CMR short axix view demonstratig myocardial late gadolinium enhancement (LGE), marked with a red arrow; E) CMR four chamber view demonstrating subendocardial and myocardial LGE marked with a red arrow and * showing the thrombus; F) CMR sagittal view demonstrating subendocardial and myocardial LGE and * showing the thrombus; G) CMR STIR short-axis view showed signal increase consistent with myocardial inflammation; H) TTE four-chamber view demonstrating a filling defect suggestive of a thrombus was detected in the LV apex.

Routine transthoracic echocardiography (TTE) revealed an ejection fraction (EF) of 55%, grade 1 diastolic dysfunction, and no evidence of ventricular pathology ( Figure B ). Coronary angiography was performed to exclude ischemic etiology, and cardiac magnetic resonance (CMR) imaging was planned to rule out myocarditis. The coronary arteries appeared normal.

Four days after the echocardiographic assessment and 10 days following the initiation of anticoagulation, CMR revealed an unexpected thrombus filling the left ventricle from the apex to the mid-ventricle ( Figure 1C and Supplementary Material 1 ). Late gadolinium enhancement (LGE) consistent with EMF was observed, with the enhancement being more prominent in the subendocardial region ( Figure 1D, E and F ). Also, signal increases consistent with myocardial inflammation and edema were observed on short-tau inversion-recovery (STIR) images ( Figure1 G ). A pericardial effusion of 17 mm was observed. Follow-up echocardiography confirmed the presence of thrombus at the left ventricular (LV) apex ( Figure 1H , Supplementary Material 2 ).

The diagnosis was EMF secondary to anthracycline chemotherapy, with intracardiac thrombus formation as a complication of EMF. Loeffler's syndrome was ruled out, as the patient's eosinophil levels remained at the lower end of the normal range. The patient was managed with antibiotic therapy and anticoagulation, and no embolic complications were observed. Unfortunately, the patient ultimately succumbed to septic shock and multile organ failure.

## Dıscussıon

Survival rates have increased with advancements in cancer therapies, but this has also led to a higher incidence of cardiac side effects associated with chemotherapy.^2^As a result, routine cardiac evaluation and monitoring of EF are recommended for cancer patients, depending on the specific chemotherapy regimen.^3^While TTE is commonly used to monitor cardiac function, CMR has proven to be more sensitive in detecting early changes in EF.^4^It can identify declines in EF before they become apparent on echocardiography and detect myocardial edema and increased extracellular volume through T1 and T2 mapping techniques. These methods can enable earlier diagnosis, often before fibrosis and LGE appear, making CMR a valuable tool in managing chemotherapy-related cardiac complications, such as the decline in LV function associated with anthracycline therapy.^5^

This case highlights a rapidly developing intracardiac thrombus in a cancer patient under anticoagulation therapy. Although LGE on MRI is typically associated with irreversible myocardial damage such as that seen in coronary artery disease, the involvement of the entire subendocardium rather than a localized lesion in the epicardial coronary artery region—along with normal coronary angiography—suggests that EMF is the primary pathology.^6^Previous studies have indicated that anthracyclines may contribute to the development of cardiac EMF, depending on the patient's treatment history.^7^

The exact trigger for thrombus formation in these patients remains unclear; however, several factors, including chemotherapy-induced cardiotoxicity, a hypercoagulable state, and the development of EMF, likely contribute to the rapid formation of intracavitary thrombus, which can lead to swift decompensation and death.^3^Therefore, clinicians must be vigilant in recognizing the multifactorial nature of the hypercoagulable state in cancer patients, as it may result in the rapid development of intracavitary thrombi.^8^

Early cardiac imaging should be considered for cancer patients presenting with newly developed cardiac or pulmonary symptoms. Intracavitary thrombus formation should be closely monitored, particularly in patients with multiple risk factors for hypercoagulation, as this can lead to significant morbidity and mortality.

Data Availability

The underlying content of the research text is contained within the manuscript.

## *Supplemental Materials

See the Supplemental Video 1, please click here .

Video 1

See the Supplemental Video 2, please click here .

Video 1
